# Accurate interdomain contacts in a mixed folded protein from NMR-guided coarse-grained simulations

**DOI:** 10.1039/d6cp01833e

**Published:** 2026-06-19

**Authors:** Billy Hobbs, Noor Limmer, Gwendolyn L. Clenshaw, Felipe Ossa, Theodoros K. Karamanos

**Affiliations:** a Department of Life Sciences, Faculty of Natural Sciences, Imperial College London UK t.karamanos@imperial.ac.uk

## Abstract

Intrinsically disordered, low-complexity regions frequently cooperate with folded domains to mediate protein–protein interactions, yet accurately describing these mixed folded–disordered systems remains challenging. To visualize these mixed folded proteins, experimentally guided coarse-grained (CG) molecular dynamics simulations are often employed to extend the timescales required to capture the complex dynamics in play. However, the minimalistic nature of these approaches often compromises structural accuracy and can lead to inaccurate inter-domain interactions. Here we introduce backbone dihedral terms directly derived from NMR chemical shift data in CG-simulations to characterize the open state of a mixed-folded construct of the anti-aggregation chaperone DNAJB6 that contains a folded J-domain and a disordered GF linker. By tuning residue-specific backbone dihedral parameters to match NMR-derived secondary-structure propensities of the linker in CG-simulations, we generate conformational ensembles that yield accurate interdomain contact maps. In agreement with analysis of NMR relaxation data, the resulting ensembles show that even in the nominally open state the linker experiences motions that resemble those of the closed state driven by hydrophobic residues in GF. More generally, we show that by expanding CG-simulations to allow them to capture both local and global structural properties, physically relevant interdomain contacts can be retrieved.

## Abstract 2

By expanding fully coarse-grained MD simulations to capture local secondary structure propensities as determined by NMR chemical shifts, accurate interdomain contact maps are generated between a disordered region and a folded domain.

## Graphical abstract

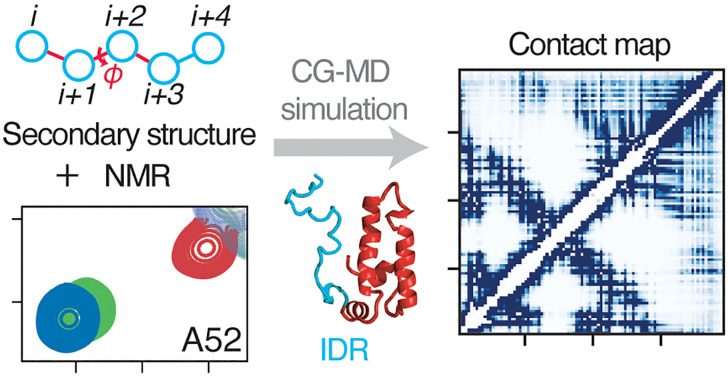

## Introduction

Low complexity regions (LCRs) in proteins have attracted significant research interest as they serve crucial roles in important biological processes.^1,2^ In many cases intrinsically disordered LCRs mediate interactions between folded domains by increasing the local concentration of the latter or by acting as hotspots for protein–protein interactions.^2^ However, and despite their importance, the atomic level characterization of low complexity regions still represents a significant task due to their inherent dynamics and similar spectroscopic signatures that complicate analysis by the majority of structural biology methods.

Computational methods have provided crucial insights into the conformational ensembles of intrinsically disordered regions (IDRs), often guided by experimental data.^3^ However, the millisecond timescales required to adequately sample IDR conformational space render all-atom molecular dynamics (MD) simulations prohibitively expensive, motivating the development of coarse-grained (CG) models.^4,5^ While CG simulations successfully capture global ensemble properties such as chain compaction, their minimalistic nature often complicates the incorporation and/or extraction of critical sequence-specific features. In particular, residue-level secondary-structure propensities—known to be functionally important in many IDRs—are difficult to represent accurately, leading to incorrect or non-physical inter-residue and interdomain contact patterns.^6^ As a result, CG approaches frequently struggle to describe the interplay between folded domains and disordered regions.^6,7^ Here, using a J-domain chaperone as a model system, we demonstrate that incorporating experimentally derived secondary-structure propensities is sufficient to recover accurate interdomain contact maps in a mixed folded–disordered protein.

The J-domain protein (JDP) family of human molecular chaperones contain a typical, low complexity, glycine and phenylalanine-rich (GF) linker that connects their J-domain with their C-terminal, substrate binding domain and is crucial for cell viability.^8^ Normally, JDPs cooperate with Hsp70 chaperones to maintain intracellular proteostasis. However, DNAJB6, a Class-B JDP, has been established as a potent inhibitor of amyloid aggregation, independent of its interaction with Hsp70.^9^ In DNAJB6, part of the GF forms a stable α-helix (helix 5 or α5) that blocks the binding of Hsp70 to the J-domain creating a closed/autoinhibited state.^10^ Even though a number of publications investigating the closed/autoinhibited state of DNAJBs are now available,^11,12^ the characterization of the open/uninhibited state is lacking. Since the GF has been implicated in various human pathologies^11^ and has also recently been shown to serve as a pseudo-substrate for Hsp70^12^ understanding its dynamics and range of motions is crucial in determining its role in JDP-mediated proteostasis. Previously, using a construct of the J-domain followed by GF in the absence of helix 5 (JD–GF) as a proxy for the open state we showed by SAXS and NMR spectroscopy that GF partially collapses against JD.^12^ Even though nominally ‘open’, the JD–GF construct shows an order of magnitude decrease in affinity for Hsp70 in comparison to JD alone, presumably due to GF–JD interactions which remain elusive in the atomic level.

In the present study we use solution NMR and CG-simulations to visualize the conformational ensemble sampled by the highly flexible JD–GF construct. Using NMR relaxation methods, we show that specific hydrophobic residues in the otherwise largely disordered GF show reduced motions in the ns timescale. To visualize the interactions between JD and GF we performed CG simulations that reproduce the overall compaction of the molecule but fail to capture the exact interdomain contacts between JD and GF. To overcome this limitation, we introduce backbone dihedral potentials that encode residue-specific secondary-structure propensities derived from NMR chemical shifts into minimal CG simulations resulting in good agreement between the simulation derived JD–GF interdomain contacts and the NMR data. Using this combined NMR and MD approach we generate a physically relevant ensemble for a disordered region in which specific phenylalanine residues in GF form extensive contacts with the J-domain resulting in partially closed states that interfere with Hsp70 binding. More generally, we show that incorporating experimentally-derived local backbone propensities provides an avenue to improving CG descriptions of disordered proteins, enabling access to atomic-level insights that are otherwise difficult to obtain.

## Methods

### Protein expression and purification

DNAJB6 JD–GF, JD–GF-α5 and DNAJB1 JD–GF constructs were expressed and purified as described previously.^12^

### Residual dipolar coupling (RDC) measurements

RDC measurements were collected at 600 MHz on a sample of 200 µM ^13^C, ^15^N-labelled DNAJB6 JD–GF in 20 mM sodium phosphate pH 7.4, 100 mM NaCl, aligned in 14 mg mL^−1^ bacteriophage Pf1 (ASLA Scientific) Backbone amide ^1^D_NH_ RDCs were measured using the ARTSY pulse sequence.^13^

### Paramagnetic relaxation enhancement (PRE) experiments

Solvent PRE experiments were performed by measuring the ^1^H *R*_2_ rate in the absence (diamagnetic sample) and presence of 5 mM TEMPO (paramagnetic sample) using a set of 6 relaxation delays ranging from 2 to 22 ms. The PRE *Γ*_2_ rate is then given by *Γ*_2_ = *R*^para^_2_ − *R*^dia^_2_.

### Spectral density mapping

Reduced spectral density mapping was performed as in ref. 14 (SI). For well-folded residues the local rotational correlation time that contains contributions from diffusional anisotropy can be obtained from the values of *J*(0) and *J*(*ω*_N_) as:1
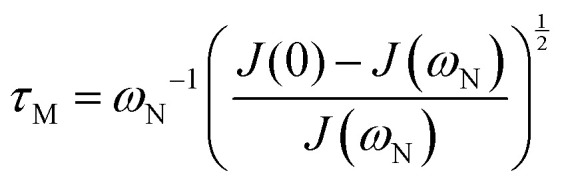


With the value of *τ*_m_ determined, the amplitude of intramolecular motion of the N–H vector can be estimated by:^15^2
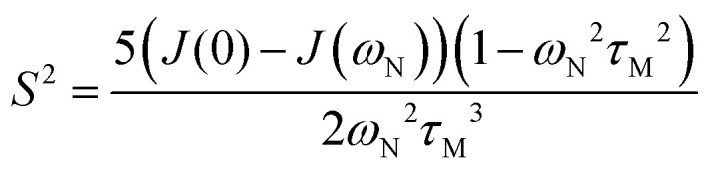


For GF residues the value of *τ*_m_ was fixed to the average value obtained for residues in JD. Given a well defined *τ*_m_ value for folded residues, eqn (2) can provide a good approximation of the generalized order parameter square (*S*^2^) for internal motions in the ns timescale. For example, *S*^2^ values obtained by eqn (2) at one magnetic field show an excellent correspondence with the ones obtained by extensive spectral density mapping at five magnetic fields for the mixed folded protein GCN4^16,17^ which shares a lot of structural and dynamic features with JD–GF.

### Model-free analysis

Model-free analysis was performed in Model-free 4.^18^ Since the closed stated in the JD–GF-α5 construct can be considered as a globular protein with a dynamic linker region, a global correlation time (*τ*_c_) was used for all residues. For the open JD–GF, a global *τ*_c_ was used for JD residues. For GF residues *τ*_c_ was fixed to the value obtained for JD. For residues in the J-domain an axially symmetric diffusion tensor was used to account for the slight anisotropy of the J-domain (*D*_||_/*D*_⊥_ ∼ 1.6). The relaxation data were fit to the following models:
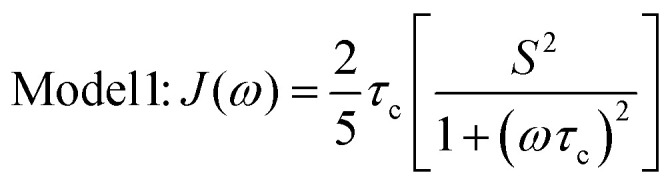

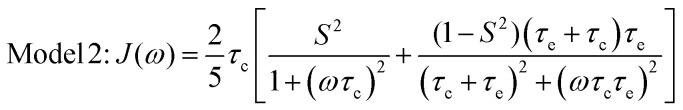

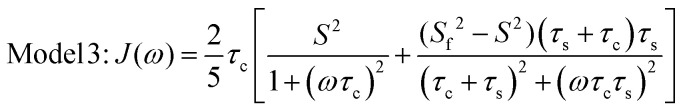


Most of the data for JD and α5 residues can be fitted to the simplest model 1 with only a few residues located in less structured loops showing significant fast motions and thus requiring the use model 2. All GF residues in JD–GF could only be fitted using the extended model-free formalism (model 3).^19^ No models that contain exchange contributions to relaxation (*R*_ex_) were used as there is no evidence of such motions in JD–GF. Model selection was performed using the Bayesian Information Criterion (BIC) and errors on the fitted parameters were obtained from 500 Monte Carlo simulations.

### Coarse-grained simulations

Martini3 simulations were performed with the Martini3IDP forcefield^20^ in OpenMM for a total of 4 (4 × 1) µs. Initial topology files were generated in martinize2^21^ followed by solvation in a 10 × 10 × 10 nm box of water using the insane script. Fully coarse-grained simulations where each residue was represented as a single bead were performed within the CALVADOS framework^5^ that uses OpenMM as its underlying engine. All simulations were repeated four times, and convergence and error estimation was performed using blocking analysis (Table S1). To allow backbone dihedral angles to be included, the HPS-SS model of Rizuan *et al.*^22^ (SI) was implemented as a C++ plugin for OpenMM and is freely available at https://github.com/karamanoslab/OpenMMDihedralPlugin. *ε*_d_ represents the residue-specific parameter that determines the probability of each residue being in a helical or extended conformation (see SI). *ε*_d_ values were determined by maximizing the agreement with the observed secondary structure propensities calculated by Talos based on the backbone Cα, Cβ, Co, N, HN chemical shifts of JD–GF. The optimized *ε*_d_ values are shown in Table S2, with a negative/positive value favoring α-helical/β-strand conformation respectively. A double well angle potential that describes the angle *θ* between three consecutive residues was also included as described in ref. 22 and 23. The addition of the dihedral angle and angle potentials has little effect on the overall compaction of the ensemble as observed earlier. Since the original HPS-SS model is parametrized for Cα dihedral angles we chose to run the CALVADOS simulations with beads centered on the Cα atom of each residue. The structure of the folded JD fully satisfies the NMR-derived dihedral angles and there is no evidence to suggest that its backbone adopts multiple conformations.^10^ Thus, the backbone potential terms were only applied to the GF linker (residues 75–98) while the JD was restrained as a rigid body using harmonic restraints. The N-terminal part of helix 3 has a slight kink picked up by its backbone chemical shifts but is fully helical in terms of its Cα configuration. The small helix formed by residues K70–N74 is too short to be picked up by Talos but has short-range ^1^H–^1^H NOEs fully consistent with a helical conformation. Default values for the stickiness parameter (*λ*) were used as described in ref. 4 and 6.

### Analysis of trajectories

Assignment of helical and extended residues in the simulations was performed as follows: if the dihedral angle *φ* between 4 consecutive beads (*i*, *i* + 1, *i* + 2, *i* + 3) falls within the helical (0.1 < *φ* < 1.5 rad) or extended (−2.5 < *φ* < −0.5 rad) well, then residues *i* + 1, *i* + 2 or *i* + 1 were assigned as pseudo-helical, pseudo-extended respectively. If three consecutive residues in a moving tripeptide window were found to be pseudo-helical/pseudo-extended, the middle residue was assigned as helical/extended. Contact order values^24^ for residue *i* were calculated as 
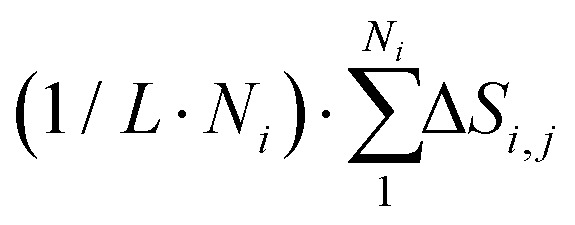
 where *N*_*i*_ is the total number of contacts for residue *i*, Δ*S*_*i*,*j*_ is the sequence separation between *i, j* residues in a contact pair and *L* is the total number of residues. Thus, the per residue contact orders reported here highlight residues involved in long range interactions. Contact order values were compared with long-range CSPs calculated as the absolute difference in ^1^H, ^15^N peak positions between the JD–GF and JD alone constructs after removing residues within 5 Å of residue G69. Principal component analysis and projection of the trajectory on the principal component vectors was performed in MDanalysis.^25^ Lifetimes for each contact pair in Martini3IDP simulations were calculated by dividing the total time that each contact was formed (defined by a distance of <8 Å) by the number of release events (defined by an increase in distance to >12 Å).^26^

## Results and discussion

### Linker dynamics by NMR relaxation

**Fig. 1:**
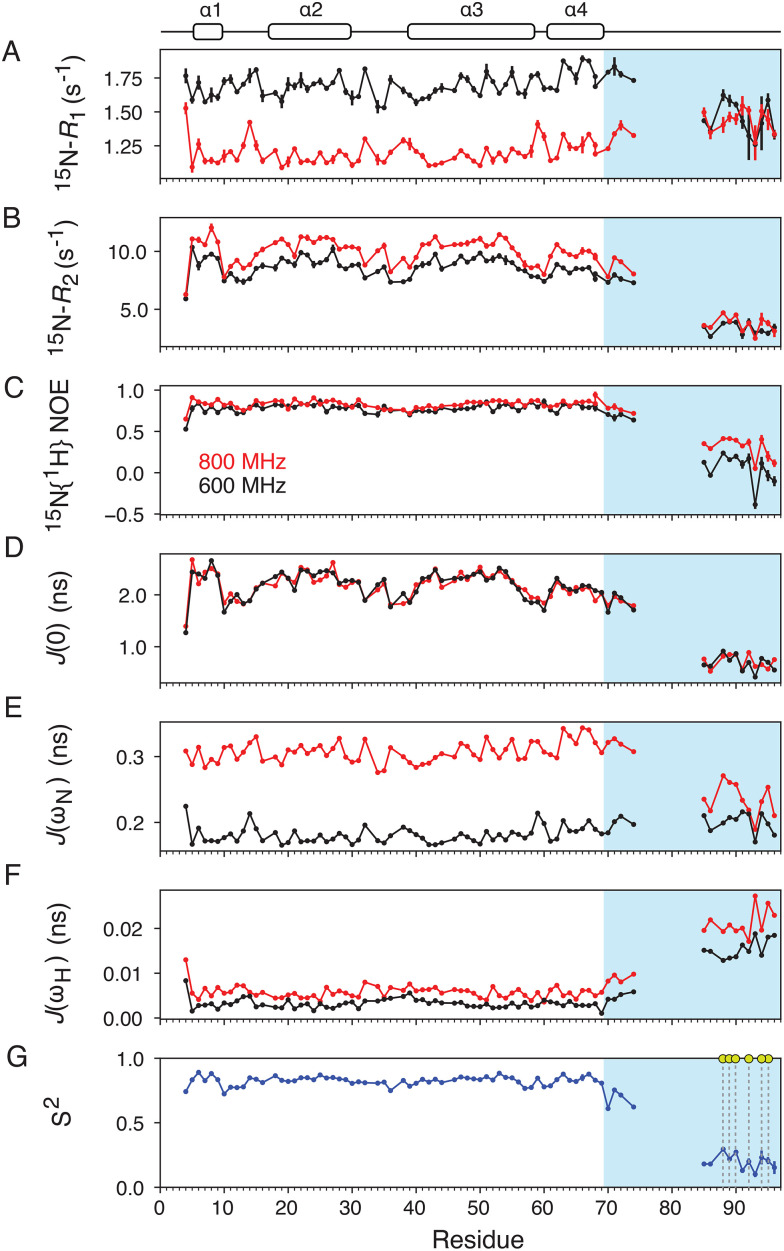
Backbone dynamics of the open JD–GF state. ^15^N *R*_1_ (A), *R*_2_ (B) and ^15^N{^1^H} NOE data (C) collected at 600 (black dots) or 800 (red dots) MHz. *J*(0) (D), *J*(*ω*_N_) (E) and *J*(*ω*_H_) (F) values are derived from reduced spectra density mapping. Calculated order parameter squared *S*^2^ from *J*(0) and *J*(*ω*_N_) at 800 MHz (eqn (2)) are shown in G. GF residues are shown as a cyan box and residues with *S*^2^ > 0.3 are highlighted with dashed lines/yellow dots.

The dynamics of the DNAJB6 JD–GF were probed by *R*_1_, *R*_2_ and NOE relaxation data at two fields and analyzed by spectral density mapping.^14^ The low complexity sequence combined with the intrinsically disordered nature of GF results in highly overlapped resonances for GF residues and therefore the relaxation data were recorded with a triple resonance HNCO readout. Raw relaxation rates shown in Fig. 1A–C are consistent with a highly ordered JD and a dynamic GF although certain residues in the latter seem to be more ordered than others. To gain more insights into the complex dynamics of the GF region the relaxation data were analysed by spectral density mapping^14^ in order to extract the values of *J*(0), *J*(*ω*_N_) and *J*(*ω*_H_) (see Methods) which are shown in Fig. 1D–F. Large *J*(0) and small *J*(*ω*_H_) values consistent with a stably folded domain are observed for JD residues as expected. Interestingly, residues K70–N74 in GF also exhibit the same behaviour of those in JD suggestive of a high degree of order for the N-terminus of GF. The patterns of *J*(0) and *J*(*ω*_H_) are reversed for the rest of GF with small and large values observed respectively in agreement with a highly dynamic region (Fig. 1D–F). *J*(*ω*_N_) values for residues D85–N93 display a rise and fall in amplitude indicative of a sequence dependent variation in nanosecond dynamics (∼1/*ω*_N_). *J*(*ω*_H_) values which report on faster, picosecond dynamics (∼1/*ω*_H_) and are not contaminated by contributions from the global tumbling of the molecule are lower for residues F87, E88, F89, F91, F93 and R94 especially at 600 MHz indicating reduced motions of these residues in the picosecond timescale. Since *ω*_H_*τ*_m_ ≫ 1 for JD–GF, *J*(*ω*_H_) values are mainly affected by internal motions and strongly depend on the order parameter squared (*S*^2^) which can be estimated purely from *J*(0) and *J*(*ω*_N_) as shown in Fig. 1G. Indeed, *S*^2^ values for F87, E88, F89, F91, F93 are all above 0.2 consistent with a smaller amplitude of motion for these largely hydrophobic residues.

To gain a more comprehensive understanding of the complex dynamics of the GF region, relaxation measurements at multiple magnetic fields can be performed and provided that the measured spectral densities are linear functions of *ω*, the amplitudes and timescales of motions at various timescales can be extracted with high precision.^17,27^ Alternatively, a more complicated model-free analysis could be performed, although this is conceptually challenging for fully disordered regions due to the absence of a single tumbling time (*τ*_c_). However, for a system like JD–GF where a relatively short IDR is tethered to and makes contacts with a well-folded domain,^12^ model-free derived motional parameters have been shown to correlate well with those from spectral density mapping at multiple fields.^17,19^ Based on previous small angle X-ray scattering (SAXS) and chemical shift perturbation (CSP) data^12^ it is safe to assume that GF and JD tumble with the same global correlation time (*τ*_c_) and thus, model-free analysis^28^ can be performed to extract the effective timescales of the internal motions. For helices 1 to 4 a simple model that takes into account the slight anisotropy of the JD diffusion tensor^29^ and only includes a single ordered parameter squared (*S*^2^) can adequately describe the relaxation data. An effective correlation time for the internal motions (*τ*_e_) on the order of 50–100 ps is only needed for loop residues in JD (Fig. 1). For GF residues however, a more complicated extended model-free formalism that includes a slow (*S*_s_^2^) and a fast (*S*_f_^2^) order parameter is required.^19^ For residues K70–N74 in the GF both *S*_s_^2^ and *S*_f_^2^ are >0.85 in the open state suggesting a remarkable rigidity of the N-terminal part of the GF-linker. These residues also show ^1^H–^1^H NOEs in the open state that are consistent with a helical structure as seen in various DNAJs (Fig. S2A).^12^ Moving towards the C-terminal part of GF, *S*^2^ values drop drastically as expected for an IDR, except for residues F87, E88 (*S*^2^ ∼ 0.50) and to a smaller extent F89, F91 and R94 (*S*^2^ ∼ 0.35) which show reduced motions in comparison to other GF residues. A good correlation is observed between the model-free and spectral density mapping derived *S*^2^ values with a Pearson correlation coefficient of 0.98 and a root-mean-square deviation of 0.06 (Fig. S1F). The average effective correlation time for the internal GF motions is on the order of 1 ns, significantly slower than the one observed for the well folded J-domain (*τ*_e_ ∼ 100 ps). Given the complex dynamics of GF, the obtained parameters are not a literal decomposition of their motions, but can be interpreted as transient, large-scale interactions which can obviously take place at various timescales but are captured by a single effective correlation time. Further support that the GF-linker does not adopt a fully random-coil structure stems from ^1^D_NH_ residual dipolar coupling (RDC) data (Fig. S2B) that show non-zero values for GF residues.

**Fig. 2:**
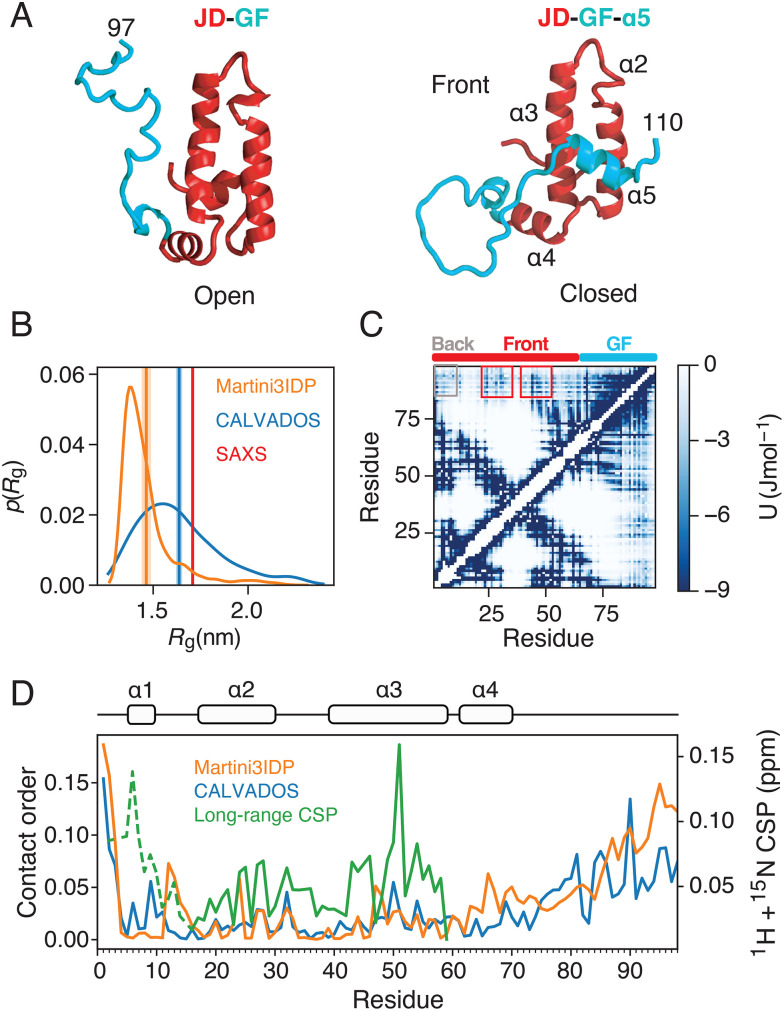
Typical CG-simulations do not reproduce long-range interactions. (A) Structures of the JD–GF (left) and JD–GF-α5 (right) (JD in red, GF in cyan). (B) Distribution of the radius of gyration from the Martini3IDP (orange) and CALVADOS (cyan) simulations with the average value and the SAXS determined value indicated as a cyan/red vertical line respectively. (C) Interaction energy maps of the Ashbaugh-Hatch term in the CALVADOS simulations. Grey/red boxes highlight the back (residues 1–15)/or the front (residues 25–30 and 45–50) of the JD. (D) Contact order values that emphasize long-range interactions for Martini3IDP (orange) and CALVADOS (cyan) overlaid with the observed long-range CSPs between JD–GF and JD alone (green) (used as a proxy for interdomain contacts, see Methods). CSPs for residues 1–15 that do not report on long-range interactions are shown as a dashed line.

It is instructive to compare the obtained motional parameters for GF residues in the context of the closed state with α5 present (JD–GF-α5 construct, Fig. S3). This construct can be considered as a globular protein with a large, disordered linker (Fig. 2A) and thus a global correlation time for all residues is appropriate and does not need to be assumed. As expected, the J-domain in the context of JD–GF-α5 behaves similarly to JD–GF (Fig. S1 and S3) with helix 5 residues also showing reduced motions consistent with a highly ordered region. GF residues still require an extended model-free formalism to explain their dynamics and show a characteristic drop-off and rise in their *S*^2^ values (Fig. S3F) with a correlation time for the internal motions of around 1.2 ns. Notably, residues K70–N74 are significantly more dynamic in the closed state showing an *S*_s_^2^ of 0.48–0.75. Taken together, analysis of the NMR relaxation data suggests that hydrophobic GF residues show reduced dynamics in JD–GF, presumably due to interactions with the J-domain.

### CG-simulations do not capture the details of the interaction between JD and GF

To structurally visualize the large-scale motions of GF we were interested in generating a conformational ensemble that captures the key properties of the mixed-folded JD–GF. In the presence of experimental data that report on long-range interactions, such as RDCs and paramagnetic relaxation enhancement (PRE) data, such ensembles have been traditionally constructed by fitting the experimental data using an ensemble approach.^30^ However, in the JD–GF-α5 strong solvent PREs are observed for GF linker residues suggesting a preferential interaction of the PRE tag with this region of the protein (Fig. S4). Thus, we specifically avoided collecting intramolecular PRE data on JD–GF since the highly hydrophobic PRE tags would likely interfere with the hydrophobic interactions that seem most likely to modulate GF motions. In theory, specific interproton NOEs between JD and GF resonances could also guide the generation of an ensemble but they require a relatively stable interaction and therefore were only observed for N- but not the C-terminus of GF (Fig. S2A). Inspired by the success of CG MD simulations for disordered proteins, we decided to take a similar approach to visualise the GF conformational ensemble. Although full-atom MD simulations are possible for systems of this size, they still require significant resources in order to achieve timescales relevant for IDRs. On the other hand, CG simulations allow sampling of the conformational ensemble of systems that vary in size in a straightforward manner. The NMR relaxation data in Fig. 1 suggested that residues K70–N74 are as rigid as those in the JD and therefore, these were fixed to their native conformations in the simulations. We tested the two most popular force-fields for IDPs, Martini3IDP which uses a multiple-bead representation and a sophisticated energy function, and CALVADOS that is fully coarse-grained with a single bead per-amino acid (Fig. S5 and S6). Martini3 that was initially developed for folded proteins^31^ samples significantly more compacted conformations (*R*_g_ 1.47 ± 0.02 nm, Fig. 2B) than those observed experimentally by SAXS (1.72 ± 0.2 nm) even with the IDP modification in its energy function (Fig. 2B and Fig. S5, S6).^20^ On the other hand, CALVADOS which relies on an empirically determined energy function but was trained directly on IDPs captures the overall compaction of JD–GF, producing an average *R*_g_ value of 1.65 nm in close agreement with SAXS (Fig. 2A and B). As seen in Fig. S7A, Martini3IDP predicts some relatively prominent long-range contacts between the back or side of the JD and GF resulting in a compact ensemble (Fig. 2B). This is in contrast with the CALVADOS simulations that produce various long-range contacts of the GF with the front and back faces of the J-domain, and also significant contacts of the GF with itself (Fig. 2C). Experimentally, by comparing the chemical shifts of JD alone *versus* those of JD–GF, we have previously shown that the GF-linker is involved in long range interactions with the front of the J-domain (Fig. S8A).^12^ To gain a more quantitative description of the long-range interactions in our simulations, we calculated contact order values for each residue, a metric that emphasizes the long-range *versus* the short-range interactions that each residue is involved in (see Methods).^24^ On top of any interdomain contacts, the observed chemical shift perturbations between the JD and JD–GF constructs contain contributions from short-range effects (such as the truncation of the protein), and possible allosteric effects. However, the latter are not expected to be significant for this system with the exception of residues 1–15 that show CSPs arising from the addition of any linker to helix 4 (Fig. S8B). Many of the residues in this area, including, Y4, V7, L8, V10 and A14 are not surface exposed (Fig. S8B), suggesting that the CSPs originate from subtle structural reorganisation of the JD domain mediated by residues in helix 4 (especially Y65) that make significant contacts with residues 1 to 15 in JD. Short-range effects can be accounted for by excluding any CSPs arising from residues in close proximity to the truncation site, resulting in a CSP dataset that reflects almost purely the long-range JD–GF interactions and can be directly compared to the contact order values calculated from our simulations. As seen in Fig. 2D, both Martini3IDP and CALVADOS simulations cannot reproduce the long-range interactions observed by NMR. Overall, CALVADOS outperforms Martini3 in sampling relevant extended conformations for JD–GF but it cannot capture the details of interdomain communication between the folded JD and disordered GF.

### Expanding CG-simulations to capture local structure

**Fig. 3:**
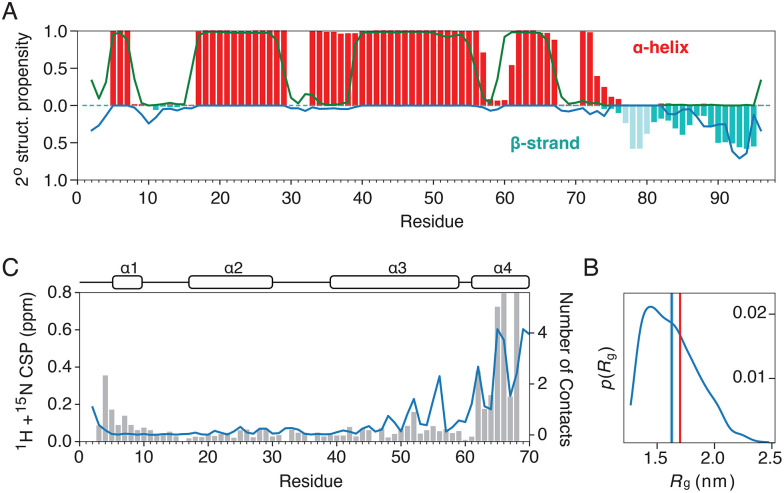
Agreement of the enhanced CALVADOS simulations with experimental data. (A) Secondary structure propensities of JD–GF calculated in the simulation are shown as bars (α-helix, red/β-strand, cyan). Talos-derived secondary structure based on the JD–GF backbone shifts are shown as lines (α-helix, green/β-strand, blue). The JD was restrained as a rigid body and backbone dihedral angles were only applied to the GF linker (residues 75–98) (see Methods). Experimental data for residues 75–80 are missing due to signal overlap of the five consecutive glycine residues. (B) Distribution of the radius of gyration from the simulations with the average value and the SAXS determined value indicated as a cyan/red vertical line respectively. (C) Combined ^1^H, ^15^N chemical shift perturbations for J-domain resonances in a JD alone *versus* a JD–GF construct. The number of contacts (<10 Å) each JD residue makes with the GF in the simulation is shown as a cyan line.

The inability of the CALVADOS forcefield to generate accurate interdomain contacts is perhaps not surprising as it was trained using experimental SAXS and PRE data that only report on the global compaction of the system.^5^ Given the good agreement with SAXS and the versatility of the fully coarse-grained CALVADOS simulations we set out to increase their ability to produce correct residue–residue contacts. To this end, we hypothesized that including new terms in the CG simulation that describe local backbone properties would allow us to generate a more accurate ensemble. The backbone chemical shifts of the GF-linker in the open state are not fully random coil but point towards a significant propensity for more extended, β-strand-like conformations.^12^ CALVADOS simulations fail to capture these local structural propensities since the underlying energy function does not contain any terms that bias the backbone towards specific conformations (Fig. S9). Capturing secondary structure propensities in single-bead CG-simulations is not straightforward as multiple beads are normally required in order to calculate backbone dihedral angles. Efforts to overcome this limitation of single bead CG-simulations have been presented before by Rizuan *et al.*^22^ who have used a double well energy potential that allows transition between an α-helical energy well to that of an extended conformation by tuning a residue specific parameter (*ε*_d_). If *ε*_d_ is set to match the known propensities for secondary structure of the various amino acids, this approach can capture the α-helical propensities for a number of IDPs.^22^ However, it is often the case that secondary structure propensities in IDRs cannot be accounted for just purely by their sequence, but are a consequence of the structural intricacies of the system as is the case for JD–GF. Therefore, we decided to tune *ε*_d_ values to match the NMR-determined secondary structure propensity of the GF-linker (Table S2) calculated from its previously determined backbone chemical shifts. In practice this approach could be useful for the plethora of IDRs that have their backbone chemical shifts determined by NMR. Including the backbone dihedral angle term with the optimized *ε*_d_ values in CALVADOS alongside a double well angle term^23^ forces GF to populate an extended conformation in its C-terminus, in good agreement with the experimental data (Fig. 3A). When compared to the ensemble that was generated without the use of the backbone potentials the calculated *R*_g_ is not significantly smaller (1.63 *versus* 1.65 nm, 2% difference) (Fig. 2B and 3B) showing that the new terms do not significantly impact the global compaction of the ensemble. As we have shown previously,^12^ the main point of contact between JD and GF is the nearby helix 4. These short-range interactions are also captured by the new simulation that shows good agreement with the experimentally determined chemical shift perturbations (Fig. 3C). Overall, the simulations can now capture both the global compaction, the local secondary structure propensities and short-range interactions of GF.

### Hydrophobic GF residues promote partially closed states

**Fig. 4:**
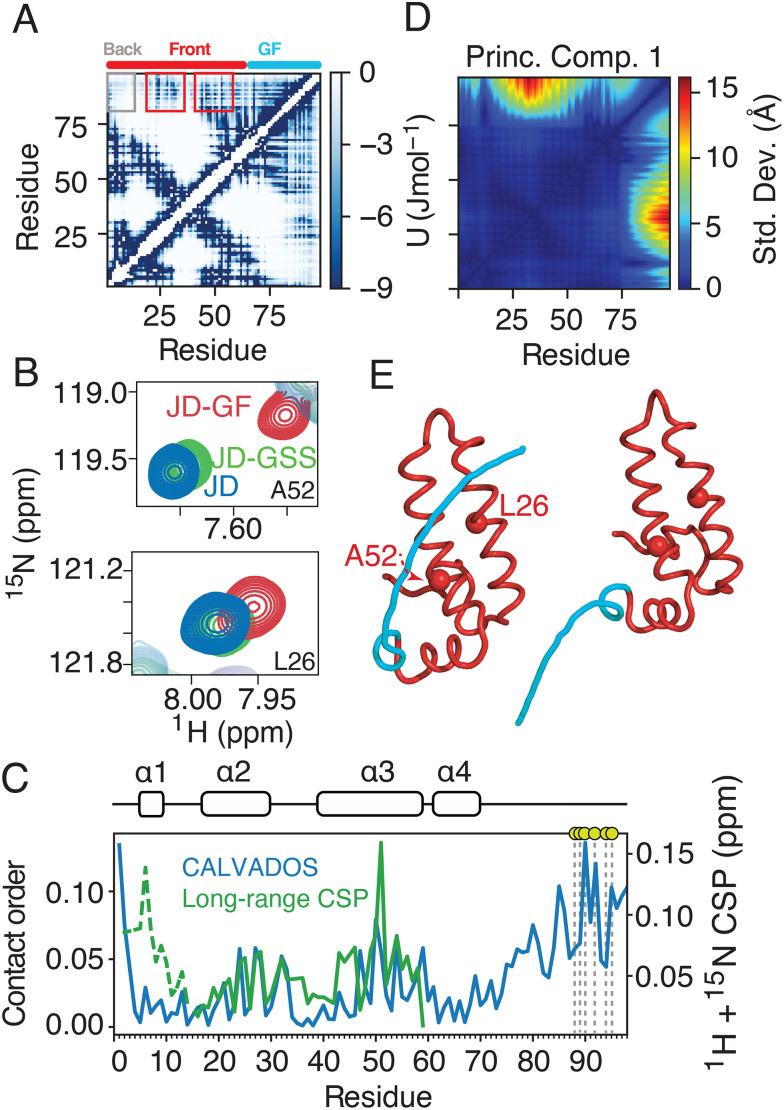
Analysis of the conformational ensemble with the dihedral term included in CALVADOS simulations. (A) Interaction energy maps of the Ashbaugh-Hatch term. Grey/red boxes highlight the back (residues 1–15)/or the front (residues 25–30) of the JD. (B) Regions of the ^1^H–^15^N of JD–GF (red), JD alone (blue) or JD-GSS (green, negative control). (C) Contact order values (cyan) that emphasize long-range interactions overlaid with the observed long-range CSPs between JD–GF and JD alone (green). CSPs for residues 1–15 that do not report on long-range interactions are shown as a dashed line. Yellow spheres and dashed lines represent the GF residues with reduced motions identified by spectral density mapping of the NMR relaxation data (Fig. 1). (D) Principal component analysis of the resulting trajectory. The principal component vectors are shown as a contact map depicting the change of each distance in relation to all other distances. Principal component 1 describes the compaction of the GF-linker onto the front face of the JD in the open state as shown in (E). L26 and A52 are shown as red spheres.

We then asked whether the new, enhanced simulations can perform better in terms of generating more accurate long-range interdomain contacts between JD and GF. As seen from the energy maps of Fig. 4A when the backbone angle potentials are included the main interdomain interactions involve exposed hydrophobic residues facing the front of the J-domain (helices 2 and 3) in good agreement with small but significant chemical shift perturbations in the same region caused by GF (Fig. 4B and Fig. S8). The new simulations show a good agreement between the calculated contact order values for residues in helices 2 and 3 that all have their side-chains facing the front of the J-domain and the long-range CSPs between JD and JF (Fig. 4C) suggesting that the introduced potential terms are able to define the correct interdomain contacts by modulating the local conformation of GF residues. Indeed, in the absence of the dihedral and angle potentials, GF partially collapses on itself and can form interactions with the nearby regions of the J-domain (Fig. 2C). However, when the GF backbone is forced to adopt an extended conformation in order to satisfy the new backbone potential terms, it can now reach further onto the front of the J-domain. Principal component analysis shows that the compaction of GF onto the front of helices 2 and 3 is the main component of the trajectory, accounting for ∼ 37% of the total motion (Fig. 4D and E). These motions of the GF relative to the J-domain create states that largely resemble the closed/autoinhibited state even under nominally open conditions, in the absence of helix 5 (Fig. 2A) rationalizing the reduced affinity for Hsc70 of the open JD–GF (∼400 µM) in comparison to JD alone (∼3 µM).^12^ Increased contact order values are also observed for several GF residues (Fig. 4C), most of which coincide with residues that show reduced motions in the ps – ns timescales as shown by spectral density mapping of NMR data in Fig. 1. It is thus intriguing to attribute the reduced motions of F87, E88, F89, F91, F93 and R94 to long-range interactions with the J-domain observed in the CG-simulations. Martini3IDP simulations can be used to estimate the lifetime of the contacts formed as shown in Fig. S7B. Although the intra-GF contacts are fast (<100 ps), some of the long-range interdomain contacts involving residues F87, E88, F89, F91, F93 are slower, on the ns timescale (Fig. S7B), as predicted by the model-free analysis in Fig. S1.

One of the most unexpected results of the analysis of the relaxation data of Fig. 1 was the apparent rigidification of residues K70–N74 in the open state in comparison to the closed one. To investigate the functional role of this collapse we removed the harmonic restraints for these residues to allow them to move freely as the rest of the GF. These new simulations show a significant reduction of the interactions between the linker and the front face of the JD (Fig. S10A) suggesting that the conformation of the N-terminal part of the linker is important in positioning the GF ensemble to the front of the J-domain. This process places the hydrophobic GF residues in a position to interact with the exposed hydrophobic residues in JD and could be important in reestablishing autoinhibition after it gets released.

In order to validate the approach and the role of hydrophobic residues in the long-range interdomain interactions, instead of performing multiple single point mutations on JD–GF we decided to swap GF with a fully disordered glycine–serine–serine (GSS) linker of the same length (JD–GSS construct) as a negative control. As seen in Fig. S10B JD–GSS shows an almost complete loss of long-range interdomain interactions in the CG-simulations. This is precisely what we observe experimentally by NMR where the chemical shift perturbations caused on JD resonances by interdomain interactions with GF have almost entirely disappeared in the presence of GSS (Fig. S8A and Fig. 4B).

## Conclusions

Here, we visualize the transient interdomain contacts between a folded domain and an IDR by integrating readily available NMR data that report on local structural features into minimal CG simulations. This NMR-driven approach is used to generate accurate interdomain contact maps for the mixed-folded JD–GF construct that lead to a significant decrease in affinity for Hsc70. Analysis of NMR relaxation data revealed reduced dynamics for specific hydrophobic residues at both the N- and C-termini of the disordered GF linker, indicating sequence-dependent interactions with the folded J-domain. By tuning residue-specific backbone dihedral parameters (*ε*_d_) to match secondary-structure propensities derived from NMR chemical shifts, accurate interdomain interaction maps between the folded domain and the low-complexity GF region can be obtained. In the absence of explicit backbone dihedral/angle terms, hydrophobic residues within the IDR preferentially collapse onto themselves or nearby sequence segments, preventing access to their native interaction interfaces on the J-domain. Enforcing experimentally determined backbone propensities promotes more extended conformations, thereby enabling these residues to engage hydrophobic surface patches that are otherwise inaccessible computationally. The resulting conformational ensembles rationalize the linker dynamics observed by NMR relaxation and reveal that the nominally open JD–GF construct samples partially closed states which resemble the autoinhibited conformation of JD–GF-α5.

Since backbone chemical shifts are typically among the first NMR observables obtained for intrinsically disordered regions, this strategy provides an experimentally accessible route to improving CG simulations of disordered proteins and mixed folded–disordered systems. The combination of NMR-driven restraints directly^32^ or indirectly (this study) into fully coarse-grained simulations is particularly attractive as it allows the generation of physically-relevant ensembles for challenging systems in a matter of minutes. We note that other force fields such as PRIMO,^33^ UNRES,^34^ HyRes^35^ and AWSEM^36^ have been designed to capture transient secondary structures in IDPs. These models achieve this through either increased backbone resolution (PRIMO, HyRes) or knowledge-based/statistical potentials (UNRES, AWSEM). In contrast, our approach introduces an explicitly tunable multiple well dihedral and angle potentials that enable direct incorporation of experimentally derived backbone conformational preferences as determined by NMR. The fact that backbone propensities can be encoded in the most minimal coarse-grained representation (single backbone bead) with the lowest computational cost allows for application of our method on systems of increasing size and complexity. For instance, regulatory IDRs in signaling proteins, transcription factors, and phase-separating or amyloid proteins are likely to exhibit similar sensitivity to local backbone properties that would be difficult to capture in full-atom simulations due to their increased size. Thus, improved CG models that can reproduce both global and local structural features of the ensemble may broadly improve the accuracy of predicted interdomain interaction maps.

## Conflicts of interest

There are no conflicts to declare.

## Supplementary Material

CP-028-D6CP01833E-s001

## Data Availability

The dihedral potential is freely available as an OpenMM plugin at https://github.com/karamanoslab/OpenMMDihedralPlugin and the raw data for the study are available at https://figshare.com/s/55a6459b127d40d02f2c. Supplementary information (SI) is available. See DOI: https://doi.org/10.1039/d6cp01833e.
