# Supplementary material for: Accurate interdomain contacts in a mixed folded protein from NMR-guided coarse-grained simulations
Source: Phys Chem Chem Phys. 2026 Jun 19;28(28):17194–202. doi: 10.1039/d6cp01833e (PMC13312411; doi:10.1039/d6cp01833e)
Supplement: CP-028-D6CP01833E-s001 [file CP-028-D6CP01833E-s001.pdf]

Supplementary Information (SI) for Physical Chemistry Chemical Physics.
This journal is © the Owner Societies 2026

Supporting Information

Accurate interdomain contacts in a mixed folded protein from NMR-guided coarse-grained simulations

Billy Hobbs[1‡], Noor Limmer[1‡], Gwendolyn L. Clenshaw[1], Felipe Ossa[1], & Theodoros K. Karamanos[1*]

Department of Life Sciences, Faculty of Natural Sciences, Imperial College London, UK

Experimental methods, 2 Supplementary Tables and 10 Figures

Supplementary methods

Spectral density mapping. The measured ^{15}N relaxation rates can be described as:

$$R_1 = \left(\frac{d^2}{4}\right)\left(3J(\omega_N) + 6J(\omega_N + \omega_H) + J(\omega_N - \omega_H)\right) + c^2 J(\omega_N) \qquad 1$$

$$R_2 = \left(\frac{d^2}{8}\right)\left(4J(0) + 3J(\omega_N) + 3J(\omega_N + \omega_H) + 6J(\omega_H) + J(\omega_N - \omega_H)\right) + \left(\frac{c^2}{6}\right)\left(J(0) + 6J(\omega_N)\right) \qquad 2$$

$$\mathrm{NOE} = 1 + \left(\frac{d^2}{4R_1}\right)\left(\frac{\gamma_H}{\gamma_N}\right)\left(J(\omega_H + \omega_N) - J(\omega_H - \omega_N)\right) \qquad 3$$

where $d = (\mu_0 h \gamma_H \gamma_N)\langle r_{NH}^{-3}\rangle$, $c = \omega_N \Delta\sigma/\sqrt{3}$, μ_0 is the permeability of the vacuum, h Planck's constant, γ_H, γ_N are the gyromagnetic ratios of ^{1}H and ^{15}N respectively, r_{NH} = 1.02 Å and $\Delta\sigma$ = -172 ppm is the chemical shift anisotropy. Reduced spectral density mapping was performed as in Supplementary reference [[1]] with values of $J(0)$, $J(\omega_N)$ and $J(\omega_H)$ are obtained as:

$$\sigma_{NH} = R_1(\mathrm{NOE} - 1)\,\gamma_N/\gamma_H \qquad 4$$

$$J(\omega_H) = \frac{4\sigma_{NH}}{5d^2} \qquad 5$$

$$J(\omega_N) = \frac{4R_1 - 5\sigma_{NH}}{3d^2 + 4c^2} \qquad 6$$

$$J(0) = \frac{6R_2 - 3R_1 - 2.72\sigma_{NH}}{3d^2 + 4c^2} \qquad 7$$

Coarse-grained simulations. The HPS-SS potential has the form[2]:

$$U_{dihed}(\varphi, \varepsilon_d) = -\ln\left[U_{dihed,\alpha}(\varphi, \varepsilon_d) + U_{dihed,\beta}(\varphi, \varepsilon_d)\right] \qquad 8$$

Where φ is the dihedral angle of four consecutive residues and $U_{dihed,\alpha}$, $U_{dihed,\beta}$ are the functions that represent the helical and extended conformations. ε_d represents the residue-specific parameter that determines the probability of each residue to be in a helical or extended conformation. The helical part is given by:

$$U_{dihed,\alpha}(\varphi, \varepsilon_d) = e^{-k_{\alpha,1}(\varphi-\varphi_{\alpha,1})^2 - \varepsilon_d} + e^{-k_{\alpha,2}(\varphi-\varphi_{\alpha,2})^4 + \varepsilon_0} + e^{-k_{\alpha,2}(\varphi-\varphi_{\alpha,2}+2\pi)^4 + \varepsilon_0} \qquad 9$$

where $k_{\alpha,1}$ = 11.4 rad^{-2}, $k_{\alpha,2}$ = 0.15 rad^{-4}, $\varphi_{\alpha,1}$ = 0.90 rad, $\varphi_{\alpha,2}$ = 1.02 rad and ε_0 =0.27. The extended part of the function is:

$$U_{dihed,\beta}(\varphi,\varepsilon_d) = e^{-\mathrm{k}_{\beta,1}(\varphi-\varphi_{\beta,1})^2+\varepsilon_d} + e^{-\mathrm{k}_{\beta,2}(\varphi-\varphi_{\beta,2}-2\pi)^2+\varepsilon_1+\varepsilon_d} + e^{-\mathrm{k}_{\beta,2}(\varphi-\varphi_{\beta,2})^4+\varepsilon_2} + e^{-\mathrm{k}_{\beta,2}(\varphi-\varphi_{\beta,2}-2\pi)^4+\varepsilon_2} \quad 10$$

where $k_{\beta,1}$ = 1.80 rad^{-2}, $k_{\beta,2}$ = 0.65 rad^{-4}, $\varphi_{\beta,1}$ = -1.55 rad $\varphi_{\beta,2}$ = -2.50, rad ε_1 =0.14 and ε_2 =0.40. ε_d values were modified from the default values to maximise the agreement with the observed secondary structure propensities calculated by Talos based on the backbone Cα, Cβ, Co, N, HN chemical shifts of JD-GF.

Supplementary Tables

Table 1: Simulation details.

Simulation #	R_g (nm)	
	**Martini3IDP**	**CALVADOS**
1	1.464 ± 0.025	1.640 ± 0.016
2	1.570 ± 0.021	1.610 ± 0.017
3	1.425 ± 0.030	1.610 ± 0.011
4	1.571 ± 0.015	1.570 ± 0.020

Table 2: Optimised ε_d values for the HPS-SS backbone dihedral potential.

Residue	ε_d
R	3.00
D	0.76
N	1.32
E	-0.58
K	0.54
H	0.11
Q	0.13
S	0.89
C	1.73
G	2.89
T	1.98
A	-1.65
M	-0.38
Y	0.94
V	0.79
W	0.37
L	-0.90
I	-0.12
P	2.65
F	0.94

Supplementary Figures

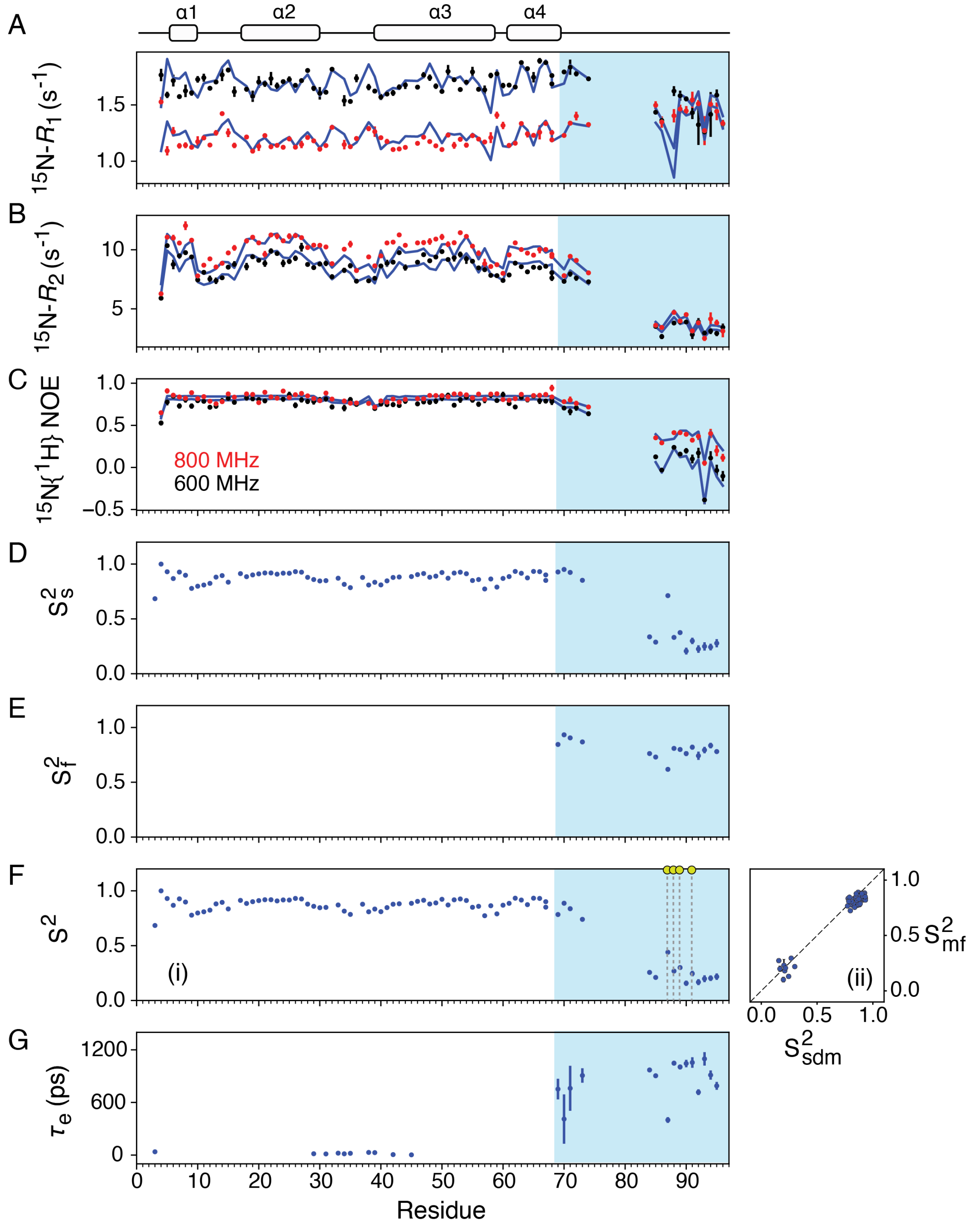

Figure S1: Model-free analysis of the open JD-GF construct. ^{15}N R_1 (A), R_2 (B) and ^{15}N{^{1}H} NOE data (C) collected at 600 (black dots) or 800 (red dots) MHz with model-free predicted values shown as blue lines. Calculated order parameter squared for the slow (S_s^2), fast (S_f^2), the generalised order parameter $S^2 = S_s^2 S_f^2$ and the correlation time for the internal motion (τ_e) are shown in D, E, F (panel i) and G respectively. Panel F(ii) shows the corelation between the spectral density-derived S^2 values with those derived from the model-free analysis (RMSD 0.06, Pearson's corelation coefficient 0.98). An axially symmetric diffusion tensor was used for JD residues and a fully isotropic extended spectral density function for those in GF. When an extended model-free formalism was used τ_e specifically refers to the slow motion ($\tau_e = \tau_s$).

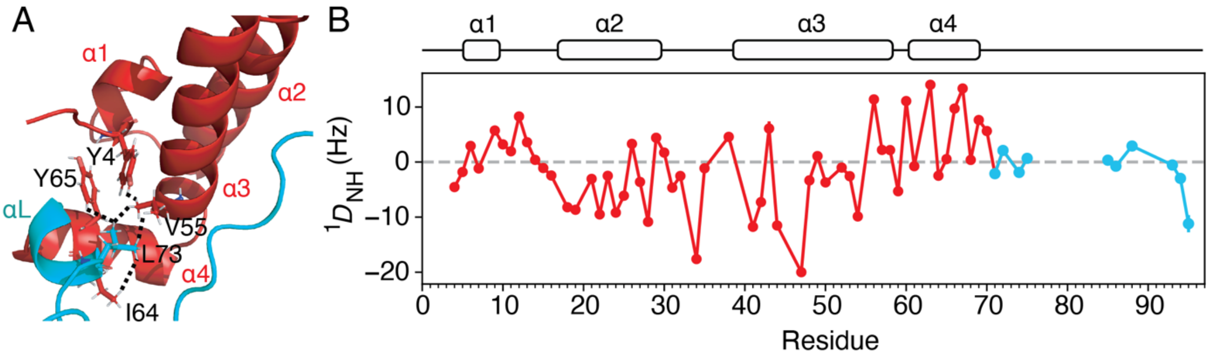

Figure S2: Structural analysis of the GF region. A cartoon representation of JD-GF with the J-domain and the GF shown in red and cyan respectively. The observed ^1H – ^1H NOEs involving residues K70 – N74 (labelled as αL) are consistent with a helical configuration of this segment (labelled as αL) and are shown as black dashed lines.[3] (B) $^1D_{NH}$ RDCs for JD-GF measured in 14 mg/mL Pf1 bacteriophage. Residues in the J-domain are shown in red and those in GF in cyan.

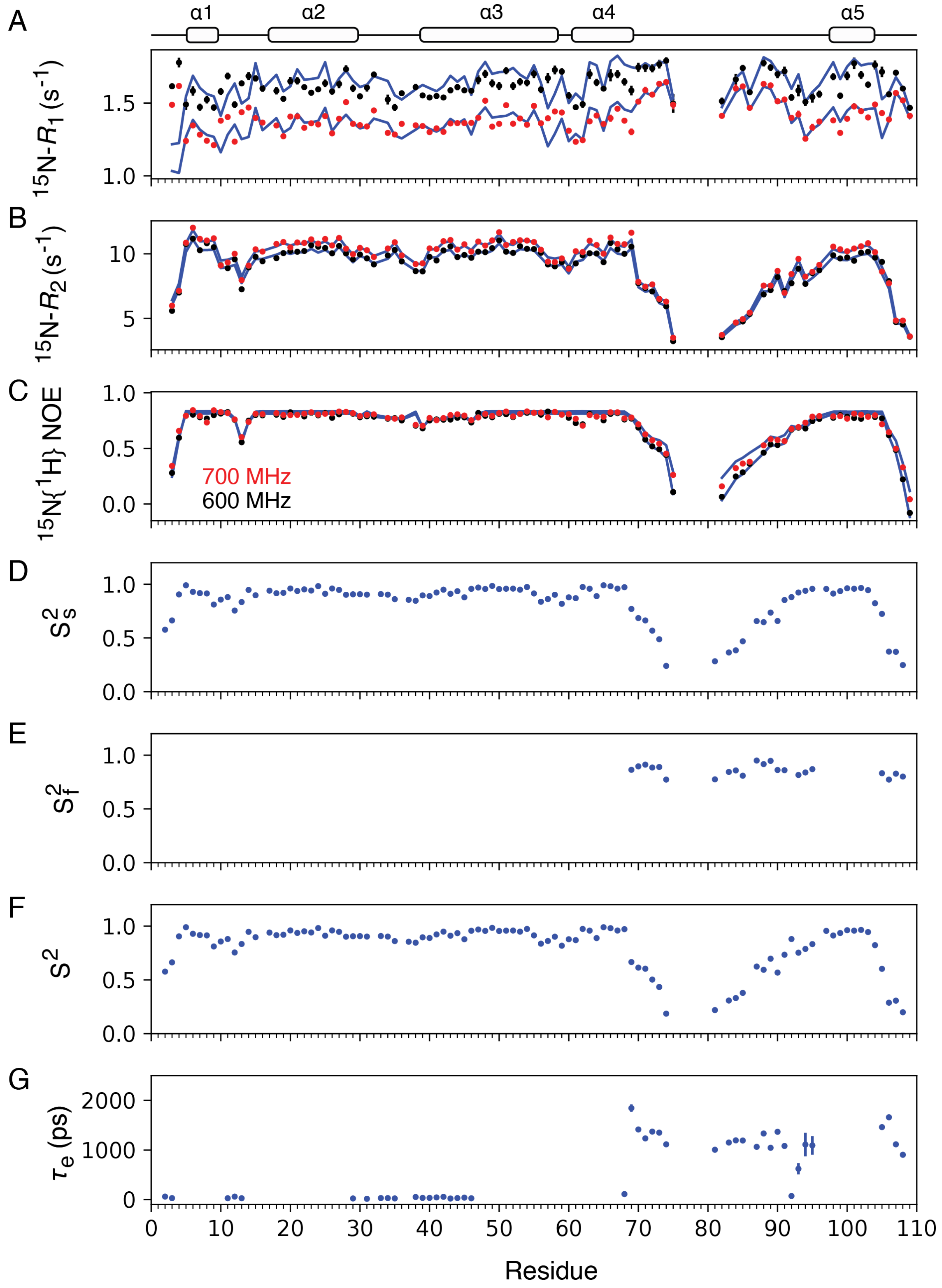

Figure S3: Model-free analysis of the closed JD-GF-α5 construct. ^{15}N R_1 (A), R_2 (B) and ^{15}N{^{1}H} NOE data (C) collected at 600 (black dots) or 700 (red dots) MHz with model-free predicted values shown as blue lines. Calculated order parameter squared for the slow (S_s^2), fast (S_f^2), the generalised order parameter $S^2 = S_s^2 S_f^2$ and the correlation time for the internal motion (τ_e) are shown in D, E, F and G respectively. An axially symmetric diffusion tensor was used for JD residues and a fully isotropic extended spectral density function for those in GF. When an extended model-free formalism was used τ_e specifically refers to the slow motion ($\tau_e = \tau_s$).

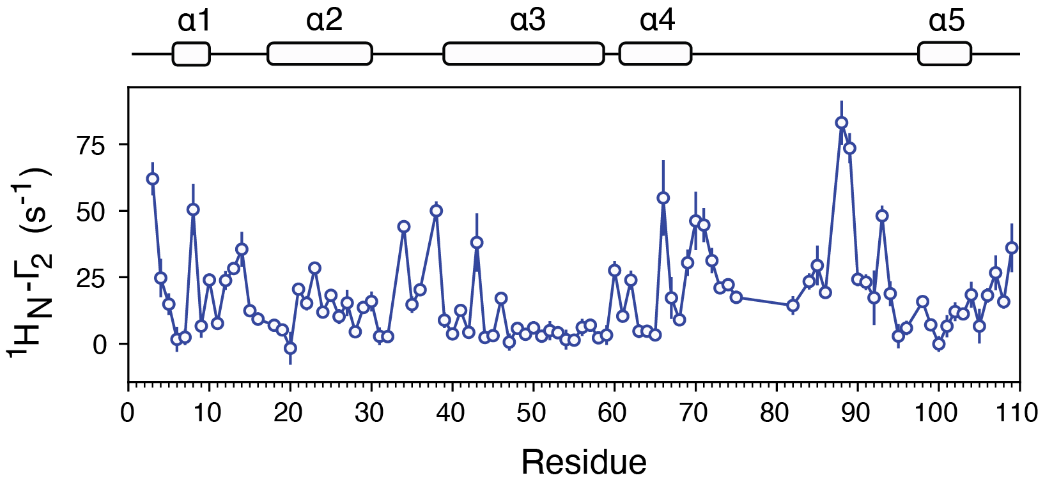

Figure S4: Solvent PREs for the closed JD-GF-α5 construct. ^1H_N PRE profiles collected in the presence of 5 mM TEMPO. Strong solvent PREs for the hydrophobic parts of the GF linker (residues 70-74 and 88-93) suggest non-specific interactions of the spin label with the GF linker.

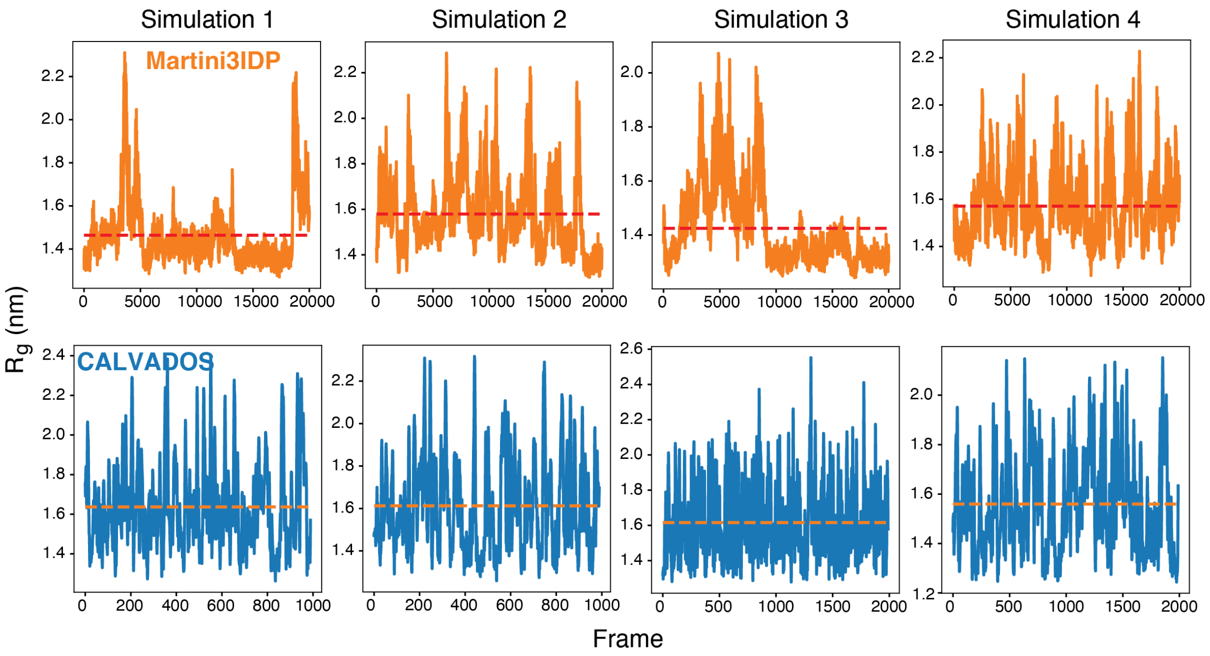

Figure S5: Time evolution of R_g. R_g as a function of frame number for the Martini3IDP (top) and CALVADOS simulations (bottom). The average R_g value for each simulation is shown as a dashed line.

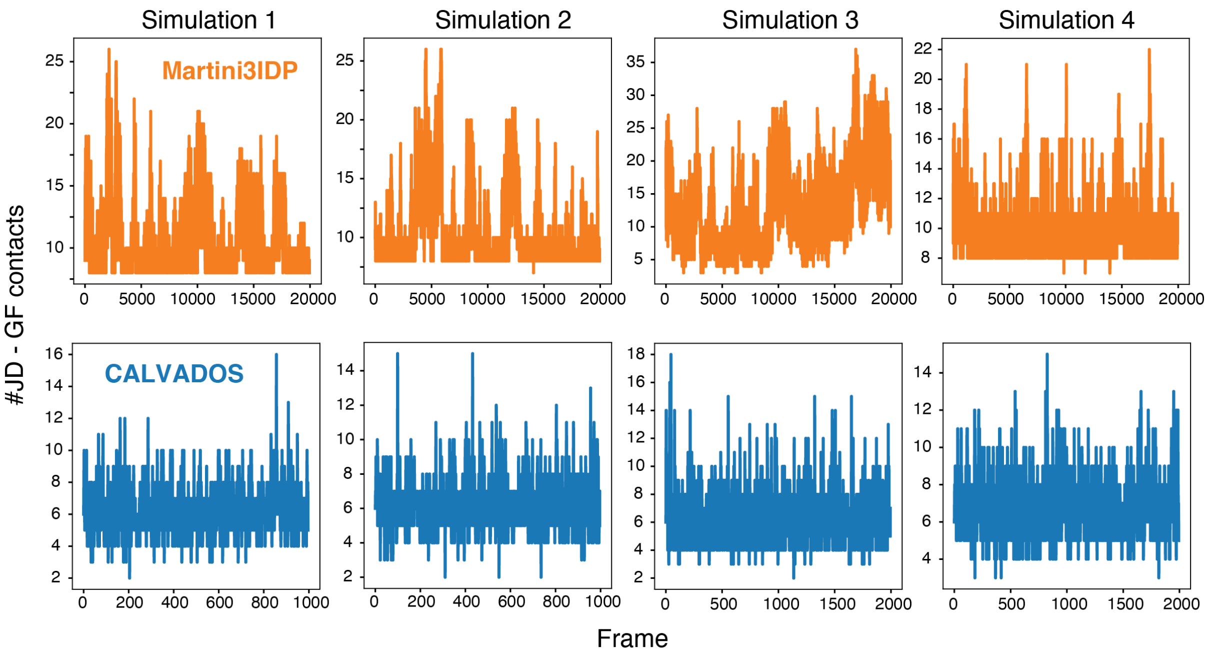

Figure S6: Time evolution of interdomain contacts. The number of JD – GF contacts as a function of frame number for the Martini3IDP (top) and CALVADOS simulations (bottom). A contact is defined when two backbone beads are within 10 Å of each other.

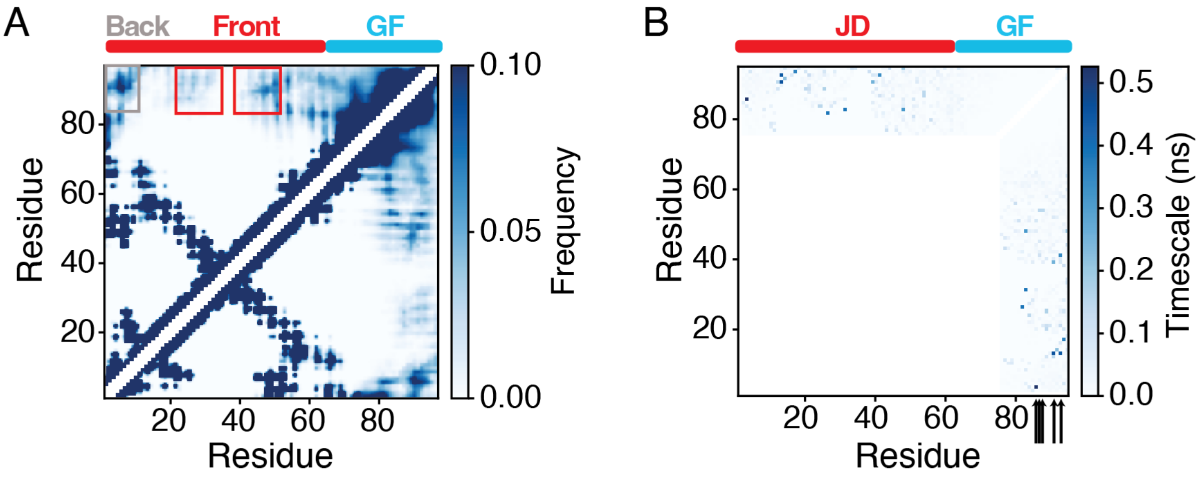

Figure S7: Contacts in Martini3IDP simulations. Maps showing the frequency (A) and the lifetime (B) of contact for each pair of residues in the concatenated 4 μs Martini3IDP simulation. The map in (A) is capped at a frequency value of 0.1 and only contacts for GF residues are shown in (B). Residues with reduced motions as identified by NMR are highlighted with black arrows in (B).

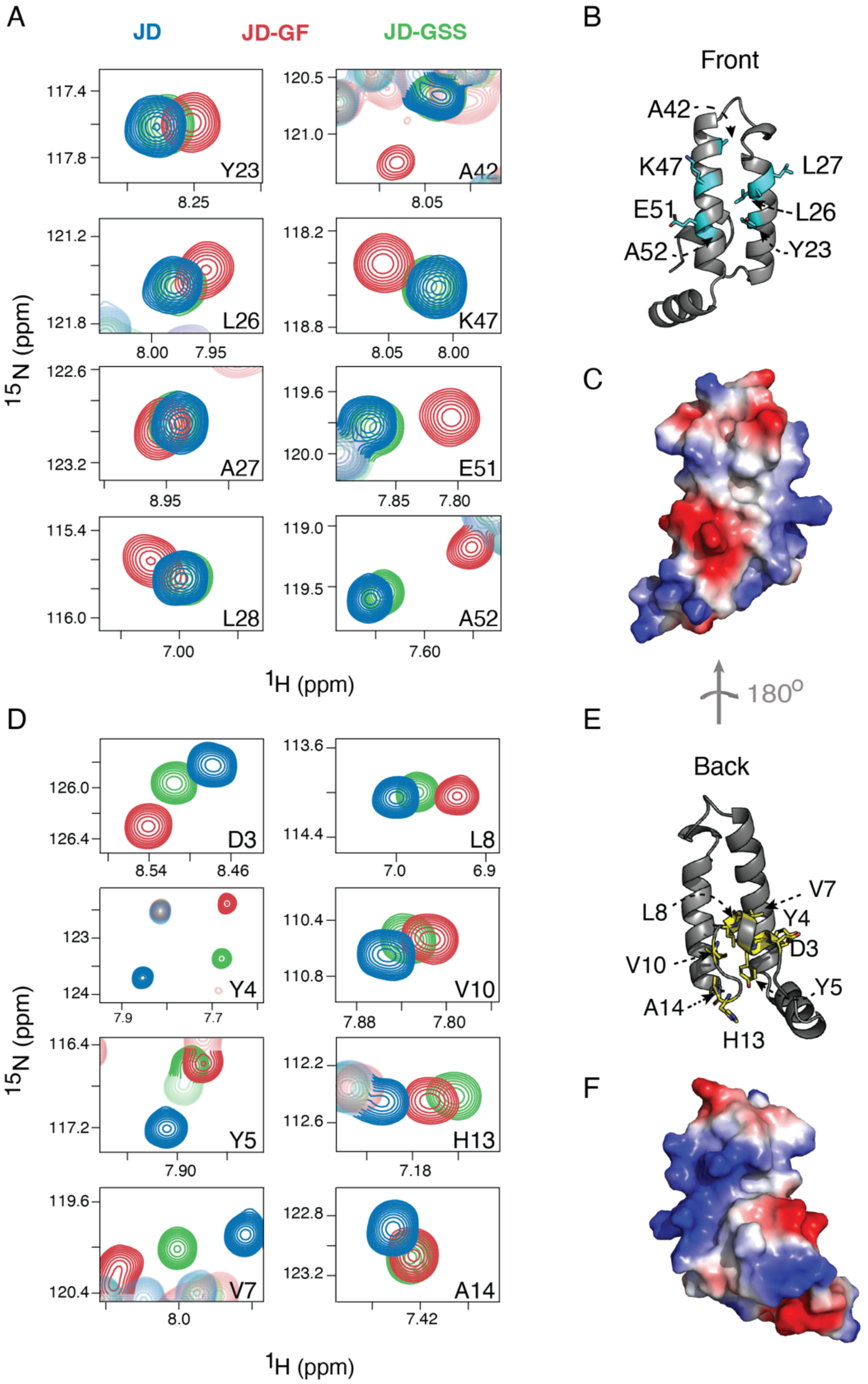

Figure S8: Contacts between the GF-linker and JD. (A) Regions of the ^{1}H-^{15}N of JD-GF (red), JD alone (green) or JD-GSS (green) for various hydrophobic, front-facing JD residues. The CSPs caused by GF essentially disappear in the presence of a disordered Gly-Ser-Ser repeats of the same length in JD-GSS. The residues highlighted in (A) are shown in cyan on the structure of JD in (B). The electrostatic surface potential of the front of JD is shown in (C) using the same pose as in (B) (red – negatively charged, white – hydrophobic, blue – positively charged). (D) As in (A) but focusing on residues 1 – 15 at the back of JD. CSPs for these residues are still present in the JD-GSS construct. Residues highlighted in (D) are shown in yellow on the structure of JD in (E) and the electrostatic surface potential of the back of JD is shown in (F).

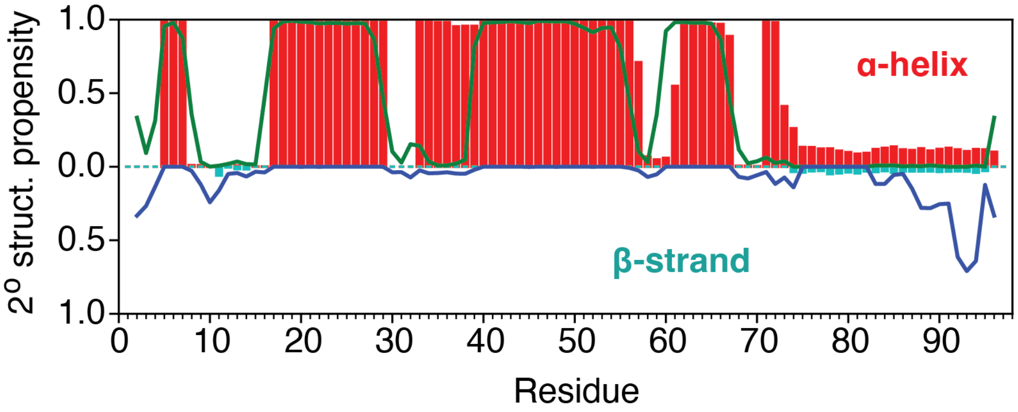

Figure S9: Conformational analysis of the JD-GF CALVADOS ensemble with no backbone dihedral angles included. Secondary structure propensities calculated in the CG-simulation are shown as bars (α-helix, red/β-strand, cyan). Talos-derived secondary structure[4] based on the JD-GF backbone shits are shown as lines (α-helix, green/β-strand, blue).

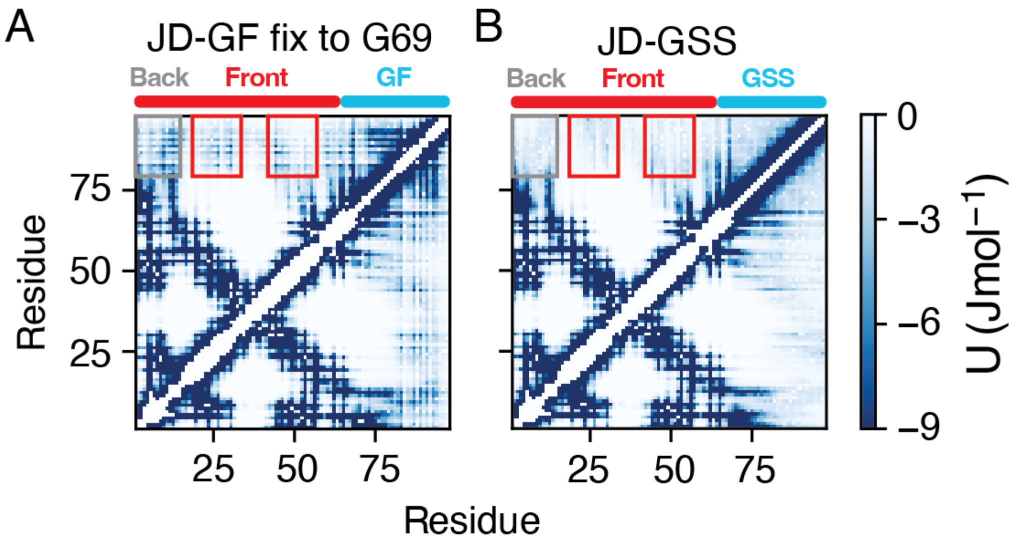

Figure S10: Modulating interdomain contacts. Energy maps of the Ashbaugh-Hatch term for a simulation where harmonic restraints were applied only up to residue G69 in JD-GF (A) or for the JD-GSS construct in which the GF is swapped with Gly-Ser-Ser repeats of the same length as GF (B). Grey/red boxes highlight the back (residues 1-15)/or the front (residues 25 - 30) of the JD.

Supplementary References

1. Farrow, N.A., Zhang, O., Szabo, A., Torchia, D.A. & Kay, L.E. Spectral density function mapping using ^{15}N relaxation data exclusively. *J. Biomol. NMR* **6**, 153-62 (1995).
2. Rizuan, A., Jovic, N., Phan, T.M., Kim, Y.C. & Mittal, J. Developing bonded potentials for a coarse-grained model of intrinsically disordered proteins. *J. Chem. Inf. Model.* **62**, 4474-4485 (2022).
3. Hobbs, B. et al. A low-complexity linker as a driver of intra- and intermolecular interactions in DNAJB chaperones. *Nat. Commun.* **16**, 5070 (2025).
4. Shen, Y., Delaglio, F., Cornilescu, G. & Bax, A. TALOS+: a hybrid method for predicting protein backbone torsion angles from NMR chemical shifts. *J. Biomol. NMR* **44**, 213-223 (2009).
